# Changes in depression, anxiety, and post-traumatic stress symptoms among children and adolescents exposed to adverse childhood experiences following participation in the PROACT intervention in Nairobi, Kenya

**DOI:** 10.3389/fpsyt.2026.1781336

**Published:** 2026-07-23

**Authors:** Teresia Mutavi, Aloyce George Mlyomi, Byron J Powell, Ozge Sensoy Bahar, Anne Mbwayo, Catherine Musyoka, Daniel Osuka, Muthoni Mathai

**Affiliations:** 1 Department of Psychiary, Faculty of Health Sciences, University of Nairobi, Nairobi, Kenya; 2 Kilimanjaro Clinical Research Institute, Kilimanjaro, Tanzania; 3 Brown School of Social Work, Washington University in St. Louis, St. Louis, MO, United States; 4 Bursky School of Public Health, Washington University in St. Louis, St. Louis, MO, United States; 5 Division of Infectious Diseases, John T. Milliken Department of Medicine, Washington University School of Medicine, St. Louis, MO, United States; 6 School of Public Health, College of Medicine & Health, University College Cork, Cork, Ireland; 7 Silver School of Social Work, New York University, New York, NY, United States

**Keywords:** ACES, anxiety, depression, LMICs, PROACT, PTSD

## Abstract

**Introduction:**

Globally, children and adolescents exposed to Adverse Childhood Experiences (ACEs) face an increased risk of developing mental health disorders. The prevalence of these mental disorders is further amplified by the lack of access to specialised mental health treatment especially in low resource settings. There is an urgent need for scalable mental health interventions that can effectively address the needs of these vulnerable populations. Non-specialist-delivered interventions, such as PROACT (Psychoeducation, Relaxation, Problem-solving, Activation, and Cognitive Coping Therapy), represent a promising scalable approach that could help bridge the existing mental health treatment gap in low-resource settings.

**Objectives:**

This study aimed to assess changes in depression, anxiety, and post-traumatic stress symptoms among children and adolescents exposed to adverse childhood experiences following participation in the PROACT intervention delivered by trained social workers in Nairobi, Kenya.

**Methodology:**

Mixed-methods pre-post study design was employed. Twenty purposively selected sites across Nairobi County each contributed one social worker (N = 20), who received training to deliver the intervention. A total of 40 children participated and received 4–6 PROACT sessions. Quantitative data were analysed using STATA version 17. Paired t-tests were used to compare baseline and endline scores, while mixed-effects linear regression models with participant ID as a random effect were fitted to estimate changes in outcomes over time and account for repeated measures. Statistically significant improvements were observed across all mental health outcomes. Mean anxiety scores decreased from 6.2 at baseline to 2.7 at endline (mean difference: −3.5; 95% CI: −4.7 to −2.2; p < 0.001), while mean depression scores decreased from 6.4 to 2.9 (mean difference: −3.6; 95% CI: −4.9 to −2.3; p < 0.001). Mean PTSD scores decreased from 16.7 (95% CI: 12.8–20.5) at baseline to 7.3 (95% CI: 4.4–10.1) at endline (mean difference: −9.4; 95% CI: −14.6 to −9.3; p < 0.001). Mixed-effects linear regression analyses corroborated these findings, demonstrating significant reductions in PTSD (β = −9.44), anxiety (β = −3.48), and depression (β = −3.59) symptoms (all p < 0.001).

**Conclusion:**

The PROACT intervention was feasible and acceptable when delivered by social workers in Nairobi primary healthcare facilities and was associated with improvements in mental health outcomes among children and adolescents. These findings highlight the potential of task-sharing approaches to expand access to mental healthcare in low- and middle-income countries (LMICs) and warrant further evaluation in controlled studies.

## Introduction

Children and adolescents exposed to adverse childhood experiences (ACEs), including physical, sexual, and emotional violence, neglect, poverty, parental mental illness, and parental loss, are at increased risk of developing mental disorders (MD) and psychosocial difficulties (1). Global statistics show that 50% of MDs begin by age 14 with one in seven (14%) of 10-19-year-olds developing a mental disorder, which accounts for 13% Global Burden of Disease (GBD) in this age group (2). Common mental health consequences associated with exposure to ACEs include post-traumatic stress disorder (PTSD), depression, anxiety, suicidality, substance use, and behavioral problems, which can negatively affect educational attainment, social functioning, and long-term wellbeing (1). Experiences such as trauma, abuse, neglect, chronic stress, and other adverse childhood experiences can substantially increase vulnerability to mental health problems during childhood and adolescence (3–5).

The COVID-19 pandemic further worsened the mental health burden among children and adolescents through social isolation, school disruptions, economic hardship, and reduced access to support systems, contributing to increased prevalence of mental disorders globally (6, 7). Despite the growing burden of child and adolescent mental disorders, access to appropriate mental healthcare remains extremely limited, particularly in low- and middle income countries (LMICs) (6). The World Health Organization (WHO) and the World Bank estimate that at least half of the world’s population lacks access to essential health services (8). As a result, a substantial treatment gap exists, with many children and adolescents receiving either no mental healthcare or they receive healthcare that is inadequate to meet their mental health needs (9, 10). A major contributor to this treatment gap is the global shortage of trained mental health professionals (9, 10). However, despite the existing need and treatment gap, only a ssmall proportion of the healthcare workforce is specialized in mental healthcare, with only 1% of the healthcare workforce globally provides mental healthcare (11).

Kenya faces a critical shortage of skilled mental health professionals, with an estimated ratio of approximately one psychiatrist per one million people. This shortage is particularly pronounced in child and adolescent mental health (12). Fortunately, evidence-based and effective psychosocial interventions are available that can help treat and prevent many mental health conditions. In response to the existing unmet need, Kenya has begun adopting task-sharing approaches to expand access to mental health services through low-intensity psychosocial interventions delivered by non-specialist providers (13–15). Social workers have increasingly been trained to provide psychosocial care and support to children and families within communities, healthcare facilities, and rehabilitation centers. This emerging model offers a practical strategy for addressing the shortage of mental health specialists and narrowing the treatment gap among vulnerable children and adolescents (16).

Building on this approach, there is a need to evaluate structured, evidence-based interventions that can be delivered by trained non-specialists. Problem-solving, Relaxation, Activation, Cognitive Coping, and Trauma-focused Strategies (PROACT) is a psychosocial intervention designed to support children and adolescents exposed to adversity and trauma. By equipping social workers with skills to deliver PROACT, access to evidence-based mental health support may be expanded while strengthening community-based care. Therefore, this study sought to assess the feasibility, acceptability, feasibility and potential clinical benefits of PROACT when delivered by trained social workers, while contributing evidence on task-sharing approaches for child and adolescent mental healthcare in Kenya.

### The intervention

The intervention, Psychoeducation, Relaxation, Problem solving, Activation, and Cognitive Coping Therapy (PROACT), is a brief 4–6 sessions (depending on severity and persistence of symptoms at the end of the fourth session), modular transdiagnostic/multi-problem intervention. PROACT builds on “Trauma Focused Cognitive Behavioral Therapy (TF-CBT)” and “Common Elements Treatment Approach” CETA implementation by lay counselors in the region (17). In PRO-ACT, clients are educated about how cognitions and behavior impact their feelings, and receive modules tailored to their individual symptom presentation and need. **Table 1** below, presents five (5) main intervention modules. The PROACT intervention was delivered to children and adolescents aged 10–17 years.

**Table 1:** Description of the study intervention.

Module	Description
Psychoeducation	Psychoeducation provides an overview of PROACT, symptoms description and their relationships to client’s situation, and begins the provider-client relationship. The provider shares what is known as common feelings and worries, thoughts and behaviors of children and adolescents.
Relaxation	In this module, children/adolescents are taught to recognize how stress is experienced in the body, learn to recognize and rate severity of stress/body tension and learning and different ways to relax the body to feel better. They are encouraged to make a table of events/feelings and practice at home, noting improvement for review with the provider.
Problem-solving	In this module children/adolescents are taught to identify and define a current problem that is causing anxiety or depression, that is in the young person’s control (e.g., they have some capacity to change or solve the problem). The social worker uses a problem-solving framework to brainstorm all possible solutions to the problem, determining the best possible solution to enact/try. In subsequent sessions, the provider and client can re-evaluate the problem and solution, repeating the cycle if need be. Provider helps the client work through internal (mood state) and external barriers to enacting solutions, and helps the client break solutions down into small steps, if needed.
Behavioral activation	In this module, social workers support the client in identifying activities they can do in the week and rating impact on mood. The social worker and child/adolescent explore possible/reachable activities that they can integrate in their day-to-day program and the client is encouraged to try those activities again.
Cognitive coping	In Cognitive Coping, clients learn that they can change their thinking to feel better (even if one cannot do something different). – the social worker explains, with examples, how thoughts, feelings, and behavior are connected and how changing thought can change the way we feel and the way we behave- referred to as “thinking differently”. Demonstrates the Thought-Feeling-Behavior triangle using real examples.

## Methodology

We conducted a mixed-methods pre–post pilot study guided by the Reach, Effectiveness, Adoption, Implementation, and Maintenance (RE-AIM) framework to evaluate the preliminary effectiveness and implementation experiences of the PROACT intervention delivered by trained social workers to children and adolescents exposed to ACEs in Nairobi, Kenya. The study aimed to assess changes in symptoms of depression, anxiety, and post-traumatic stress following participation in PROACT, while also exploring implementation-related factors that could inform future scale-up and integration within community and primary healthcare settings.

Participants were recruited through an existing community referral pathway. Community Health Promoters (CHPs) identified children and adolescents with a history of ACE exposure within their communities and referred them to social workers at participating health facilities. Social workers subsequently notified the study team, which conducted eligibility screening and enrolment assessments. A total of 73 children and adolescents referred through this pathway were screened for eligibility. Participants were eligible if they were aged 10–17 years, had experienced one or more ACEs, screened positive for symptoms of depression, anxiety, or post-traumatic stress, were able to participate in psychosocial sessions, and provided assent alongside parental or caregiver consent where applicable. Participants with severe depressive symptoms requiring specialist mental healthcare were excluded and referred for further psychiatric assessment and management.

Of the 73 individuals screened, 46 met eligibility criteria. Twenty-five did not meet symptom eligibility criteria because they did not report clinically significant symptoms of depression, anxiety, or post-traumatic stress, while two participants presenting with severe depressive symptoms were excluded and referred for specialist care. Of the 46 eligible participants, six were not enrolled because four returned to boarding school and two relocated from the study area before intervention initiation. Consequently, 40 children and adolescents were enrolled and received the PROACT intervention.

Quantitative data were collected at baseline and immediately following intervention completion (4–6 weeks) using standardized mental health assessment tools. Participants received individualized PROACT sessions tailored to their psychosocial needs. All 40 participants completed the intervention and endline assessment and were included in the final quantitative analysis.

To complement effectiveness outcomes, implementation-related experiences of intervention delivery and uptake were explored qualitatively through interviews with social workers and healthcare providers involved in implementation.

### Qualitative component

Qualitative interviews were conducted at the end of the intervention period among social workers who delivered the intervention and selected healthcare providers and facility in-charges working within participating primary healthcare facilities in Nairobi. A total of six key informant interviews were conducted. Data collection ceased when thematic saturation was observed across interviews, with later interviews largely reinforcing previously identified concepts and yielding limited new implementation-related insights. Thematic saturation was assessed through ongoing review of interview transcripts and comparison of emerging codes and themes during the concurrent data collection and analysis process.

Participants were purposively selected based on their direct involvement in the delivery, supervision, or oversight of the PROACT intervention, enabling the collection of rich, experience-based perspectives on implementation processes and outcomes. Interviews explored participants’ perceptions of the intervention, including its acceptability, feasibility, integration into routine service delivery, perceived benefits for children and adolescents, and considerations for sustainability and future scale-up. Data were collected using a semi-structured interview guide informed by the Reach, Effectiveness, Adoption, Implementation, and Maintenance (RE-AIM) framework. The guide explored experiences with intervention delivery, facilitators and barriers to implementation, perceived participant outcomes, organizational support, and recommendations for future implementation. All interviews were conducted in English by trained members of the research team, audio-recorded with participants’ consent, and transcribed verbatim for analysis.

Qualitative data were analyzed using reflexive thematic analysis. Analysis initially followed an inductive approach, allowing codes and patterns to emerge from participants’ accounts rather than being imposed *a priori*. Two members of the research team (AGM and DO) independently read and re-read the transcripts to familiarize themselves with the data and conducted line-by-line coding. Through an iterative process of comparison and discussion, an initial coding framework was developed and refined as new concepts emerged across interviews. Coding differences were discussed and resolved through consensus meetings involving a third researcher (TM), ensuring that interpretations accurately reflected participants’ perspectives. Following coding, related codes were grouped into broader categories and subsequently synthesized into higher-order themes representing key implementation experiences. The resulting themes were then mapped deductively to the RE-AIM framework to facilitate interpretation and reporting of implementation outcomes. This hybrid inductive–deductive approach enabled findings to remain grounded in participants’ experiences while also being situated within an established implementation science framework.

### Ethical considerations

This study received research approval from University of Nairobi/KNH Ethics and Research Committee and NACOSTI and was assigned the research registration number **P632/08/2024**, subsequently, we obtained permission to proceed with the study from the Nairobi City County authority. At the health facility level, we sought permission to conduct our research. For participants, including children, healthcare providers, and social workers, we clearly explained the main objective and specific purpose of the study, as well as the potential benefits and risks associated with participation, and addressed any questions they had about the study. Participants who were willing to take part were also informed of their right to voluntarily withdraw at any time during the study without any repercussions. We therefore requested that they provide informed consent by signing a consent form. Only then did we proceed with the study. Participants who had severe symptoms or safety concerns were referred to clinician in the facility. No adverse events were reported throughout the duration of the study among study participants.

### Study-setting

Our study was conducted in Nairobi County, which comprises 17 sub-counties (Makadara, Starehe, Mathare and Embakasi North, South, East, West and Central, Westlands, Dagoreti North and South, Langata, Kibra, Kamukunji, Kasarani, Ruaraka) and a population of approximately 4,397,073 according to the 2019 Population and Housing Census. Nairobi is one of Africa’s major capital cities with a large population, including many children facing mental health challenges and ACEs, stemming from the harsh living conditions experienced by lower- and middle-income populations, who constitute about 60-80% of Nairobi’s total urban population (18). The intervention was piloted in twenty health facilities located within the sub-counties of (Makadara, Mathare and Embakasi North, South, East, West and Central, Westlands, Dagoreti North and South, Langata, Kibra and Kamukunji). These sub-counties were selected because they have large populations from lower-income groups and a high prevalence of ACEs among children, this being the primary reason for selecting health facilities in these areas.

### Sample determination and selection

**Figure 1:**
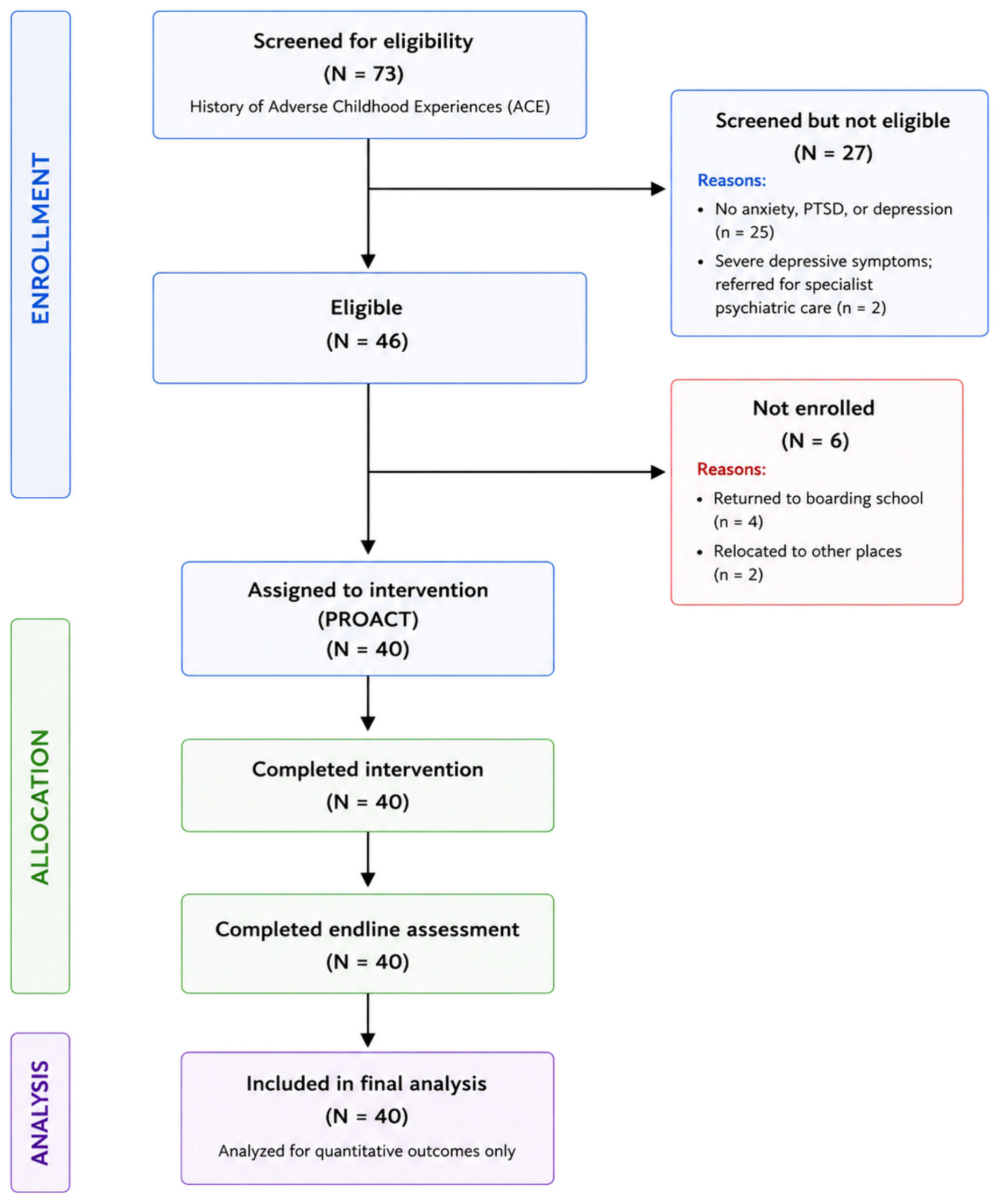
Participant flow diagram for the PROACT intervention among children and adolescents exposed to adverse childhood experiences in Nairobi, Kenya.

We began by identifying all Level 4 health facilities in Nairobi, Kenya. A total of 71 facilities were listed and then grouped into clusters based on workload. Following this, we randomly selected a total of 20 level 4 facilities for data collection and intervention piloting. The recruitment process for all human participants involved, first identifying potential study participants based on their professional role (for social workers and healthcare providers) and history of ACEs (for children). A total of twenty (20) social workers (2 males, 18 females from the selected hospitals were purposively recruited and trained to participate in the study as intervention providers. Forty-six (46) children with a history of ACEs from any of the recruited health centers were recruited using convenience sampling method into the study. Of these, 40 children completed the intervention until the end, 6 children withdrew from the intervention without any complications either due to relocation in the village, and going back to boarding school. The characteristics and outcomes of those who withdrew were not significantly different from those who completed the intervention. **Figure 1** shows the participant flow through the study.

### Training of research assistants and intervention deliverers

Two research assistants (clinical psychology students from the University of Nairobi) were recruited to participate in the research process. Both were trained for a period of two days on the ethical principles of conducting human subjects research, as well as ethical data collection and handling procedures. The training placed special emphasis on conducting research with minority populations, particularly children adversely affected by ACEs. The training was conducted in person and facilitated by two PhD-level senior researchers (AM and TM) from the University of Nairobi. This training equipped the research assistants with the skills to ethically interact with children and screen for ACEs, depression, anxiety, and Post-Traumatic Stress Disorder (PTSD).

### Intervention deliverers

All recruited social workers underwent five days of training on the PROACT Intervention. The training was conducted in person, from 8:00 AM to 4:00 PM daily. A total of five (5) certified PROACT trainers and supervisors facilitated the training, two holding PhDs and three holding bachelors degrees.The training curriculum covered the following topics 1; identifying children with mental health challenges, including depression, anxiety, and post-traumatic stress disorder 2; understanding and application of relaxation techniques 3; understanding problem-solving skills 4; applied behavioral activation skills 5; understanding and applying cognitive coping techniques. Following the training, all social workers underwent two weeks of virtual supervision through simulated case management. In this process, every two social workers were paired and assigned simulated case scenarios in which one social worker enacted the role of the client while the other managed the case, all under observation by the trainers to assess their competence in delivering the PROACT intervention.

### Measures

All children’s involved in this study, were assessed for ACEs, depression, anxiety and PTSD using standardized tools during pre and post intervention: the tools are presented in **Table 2**.

**Table 2:** Brief description of the study instruments.

No	Tool	Brief description
1	The WHO ACEs International Questionaire	This is a 13 item self-report questionnaire used to assess the ACE types and scores (WHO, 2018).
2	The Child PTSD Symptom Scale self-report (CPSS-SR) for DSM-5	The CPSS-SR-5 scale is a 27-item scale with the first 20 items rated on a 5-point Likert scale depicting frequency and severity of symptoms from zero to four while the last seven are functioning items with a yes or no response and these depict the effect of symptoms on daily functioning.
3	Patient Health Questionnaire 9 (PHQ-9) Screener adolescent version	This is a nine-item screener that will be used to screen for depression and the severity cutoffs will be a total score of 5–9 for mild depression, 10–14 for moderate depression,15–19 for moderately severe depression and 20–27 for severe depression (Mansour et al., 2020).
4	Generalized Anxiety Disorder Screener (GAD -7)	This is a 7-item scale that will be used to assess for Anxiety and the cut offs that will be used will be 5–9 for mild anxiety, 10–14 for moderate anxiety and 15–21 for severe anxiety.

### Statistical analysis

We used descriptive statistics to summarize the demographic characteristics of our study participants and visualised them on graphs and percentages. Mixed effect linear regression was used to estimate the improvement following the intervention while accounting for repeated measures within individuals among participants measured at both baseline and endline (post-intervention). To assess improvement following the intervention, we examined within-participant changes in symptom scores for depression (depression symptom scores), anxiety (anxiety scores), and post-traumatic stress disorder as measured by the Child PTSD Symptom Scale (CPSS) scores. Analysis was conducted using STATA software, version 17. Regression coefficients (β) and p-values were reported, with statistical significance set at p < 0.05 (two-tailed), and results presented with 95% Confidence Intervals (CIs). Secondly, the study employed Wilcoxon signed-rank test to estimate median change scores and corresponding IQRs. The magnitude of change was quantified using the effect size (r), calculated as *z/√N*, where *z* is the standardized test statistic and *N* is the sample size. Proportions of participants showing improvement (reduction in symptom score from baseline) were also reported. All statistical tests were two-tailed, and p-values < 0.05 were considered statistically significant.

## Results

**Table 3** shows the description of the study respondents. The study recruited 40 participants, comprising of 30 females and 10 males. The mean age of all participants was 14.2 years (SD = 2.88). When disaggregated by gender, females had a higher average age of 15.12 years (SD = 2.50), compared to males, whose mean age was 11.45 years (SD = 2.14). The difference in mean age between females and males was 3.67 years, with a standard error of 0.62. The majority of those recruited identified as Christian (85%), with a higher proportion among females (93.3%) than males (60%). Most participants reported living with their parents (76.3%), while smaller proportions lived with guardians (15.8%), a spouse (5.3%), or a stepparent (2.6%). Majority (70%) of the participants were in primary school. Regarding the marital status of parents, 40% of participants had separated parents, 25% had parents who were living together, 25% were from single-parent households, and 10% had divorced parents.

In terms of parental survival, 77.5% of participants reported that both parents were alive, while 22.5% had lost one parent. Socially, 90% of the respondents indicated that they had close friends at school, with all male participants affirming this. Educational attainment among parents or guardians was generally low; half of the participants (50%) reported that the highest level of education attained by their parents or guardians was primary school, 37.5% reported secondary school, and only 10% reported college or tertiary education. A small proportion (2.5%) were unsure of their parents’ educational level.

With respect to household income, most participants (75%) came from single-income families, while 22.5% were from two-income households. Only one participant (2.5%) reported coming from a family with no income. Housing conditions varied, with nearly half (45%) of the participants living in metal housing units, followed by 30% in brick houses, 20% in earthen structures, and 5% in wooden dwellings. The socio-demographic characteristics are as summarized in **Table 3** below. Among the 40 participants included in the analysis, half (50%) reported experiencing a high burden of adverse childhood experiences (ACEs), defined as four or more events. This high ACE burden was more prevalent among females, with 60.0% reporting ≥4 ACEs compared to only 20.0% of males. Conversely, a low ACE burden (<4 ACEs) was reported by 80.0% of males and 40.0% of females.

**Table 3:** Socio-demographic characteristics of respondents (N = 40).

Characteristic	n	%
Religion		
Christian	34	85
Muslim	6	15
Whom do you live with		
Guardian	6	15.8
Parents	29	76.3
Spouse	2	5.3
Stepparent	1	2.6
What is your level of education?		
Primary school	28	70.0
Secondary school level?	12	30.0
What is the marital status of your parents?		
Divorced	4	10
Living together	10	25
Separated	16	40
Single	10	25
Parents both alive?		
Both alive	31	77.5
One alive	9	22.5
Do you have close friends at school?		
No	4	10
Yes	36	90
What is the highest level of education attained by your parents/guardians?		
College/Tertiary School	4	10
Don't Know	1	2.5
Primary School Level	20	50
Secondary School Level	15	37.5
In terms of income in your family, is your family		
A no income family	1	2.5
A single income family	30	75
A two-income family	9	22.5
What is the type of housing unit you live in?		
Brickhouse	12	30
Earthen	8	20
Metal	18	45
Wooden	2	5
ACES		
Low ACE burden (<4)	20	50
High ACE burden (>4)	20	50

### Changes in mental health symptoms following the intervention

Mental health outcomes were assessed using the Patient Health Questionnaire-9 Adolescent Version (PHQ-A), Generalized Anxiety Disorder-7 (GAD-7), and Child PTSD Symptom Scale (CPSS-SR-5). Higher scores indicate greater symptom severity across all measures. For the PHQ-A, scores of 5–9 indicate mild depression, 10–14 moderate depression, 15–19 moderately severe depression, and ≥20 severe depression. For the GAD-7, scores of 5–9 indicate mild anxiety, 10–14 moderate anxiety, and ≥15 severe anxiety. For the Child PTSD Symptom Scale (CPSS-SR-5), scores of 31–33 or higher are commonly used to indicate probable PTSD.

**Table 4** shows changes in mental health outcomes following the intervention among 40 participants using both unadjusted and adjusted analyses. At baseline, the mean Child PTSD Symptom score was 16.7 (95% CI: 12.8, 20.5), which significantly declined to 7.3 (95% CI: 4.4, 10.1) at endline. The unadjusted mean difference was 9.4 points (95% CI: 9.3, 14.6; p < 0.001). Mixed-effects linear regression, adjusting for sex and baseline severity and accounting for repeated measures by including a random intercept for participant ID, confirmed a significant reduction in PTSD symptoms (β = −9.44, 95% CI: −13.40, −5.48; p < 0.001).This finding indicates lower levels of trauma-related symptoms at endline compared with baseline among study participants.

**Table 4:** Change in mental health outcomes from baseline to endline (N = 40).

Outcome	Baseline mean (95%CI)	Endline mean (95%CI)	Mean difference (baseline mean-endline mean)	Mixed effects regression β (CI)
PTSD	16.7(12.8,20.5)	7.3(4.4, 10.1)	9.4 (9.3, 14.6) **	-9.44(−13.40, −5.48) **
Anxiety	6.2(5.2,7.1)	2.7(1.8, 3.6)	3.5(2.2, 4.7) **	-3.48(−4.67, −2.30) **
Depression	6.4(5.5,7.3)	2.9(1.9, 3.8)	3.6(2.3, 4.9) **	-3.59(−4.73, −2.46) **

Mean differences represent unadjusted pre-post changes. Mixed-effects regression coefficients reflect adjusted estimates controlling for sex and age, with participant ID as a random effect, CI- confidence interval, **p < 0.001.

Similarly, mean anxiety scores decreased from 6.2 (95% CI: 5.2, 7.1) corresponding to the mild anxiety range on the GAD-7 to 2.7 (95% CI: 1.8, 3.6), yielding an unadjusted mean difference of 3.5 points (95% CI: 3.7, 5.2; p < 0.001). The adjusted model showed a significant decline in anxiety symptoms (β = −3.48, 95% CI: −4.67, −2.30; p < 0.001).

Depression severity reduced, with depression scores dropping from 6.4 (95% CI: 5.5, 7.3) consistent with mild depressive symptoms at baseline to 2.9 (95% CI: 1.9, 3.8) indicating minimal depressive symptoms at endline, with an unadjusted mean difference of 3.6 points (95% CI: 2.3, 4.9; p < 0.001). After adjustment, the regression coefficient remained significant (β = −3.59, 95% CI: −4.73, −2.46; p < 0.001). Although the average baseline PCL-5 score was below the screening threshold for probable PTSD, the substantial reduction observed at endline suggests meaningful improvement in trauma-related symptoms among participants.

The Wilcoxon signed-rank test method was further used to detect find within-group improvement, for all participants who took part in this study. Consistent to the regression findings show in **Table 4** above, the Wilcoxon signed rank test results in **Table 5** below showed that depressive symptoms mean scores dropped from a mean of 7 (IQR 4.5–8) at baseline to 2 (0–4.5) at endline. The median change of −4 points (IQR −7 – −2) was statistically significant (z = 4.37, p < 0.001), corresponding to a large effect size (r = 0.69). These findings indicate lower depressive symptom scores at endline relative to baseline among study participants.

**Table 5:** Changes in mental health outcomes using the Wilcoxon Signed rank test.

Outcome	Baseline (Med, IQR)	Endline (Med, IQR)	Change (Med, IQR)	z-value	p-value	Effect Size	Improved n (%)
Depression	7 (4.5–8)	2 (0–4.5)	−4 (−7 – −2)	4.37	<0.001	0.69	33 (82.5%)
Anxiety	7 (4–8)	2 (0–3)	−3.5 (−7 – −1)	4.16	<0.001	0.66	31 (77.5%)
PTSD	20 (2–26)	4.5 (0–9.5)	−11 (−18 – 2)	3.63	<0.001	0.57	25 (62.5%)

IQR, Interquartile Range; Med, median; Effect Size = interpreted within Cohen’s d thresholds.

Whereas the overall change was −4 (−7 – −2), which was statistically significant (Z-score 4.37, p <.001), with medium effect size (d = 0.69). Similarly, for anxiety symptoms, changes in GAD-7 scores from a baseline score of 7 (4–8), to an endline score of 2 (0–3), were recorded with an overall change of −3.5 (−7 – −1), which was also significant (Z-score 4.16, p <.001), with medium effect size of 0.66. The median anxiety score shifted from the mild anxiety range at baseline to the minimal anxiety range at endline.

For post-traumatic stress symptoms (PTSD), median scores dropped from 20 (IQR 2–26) at baseline to 4.5 (IQR 0–9.5) at endline, yielding a median change of −11 points (IQR −18 – 2) (z = 3.63, p < 0.001; r = 0.57). Participants generally reported lower trauma-related symptom scores at endline than at baseline. Overall, 82.5% of participants demonstrated improvement in depressive symptoms and about 77.5% of adolescents reported lower anxiety scores at follow-up.

### Qualitative findings

A total of six interrelated themes were identified through thematic analysis, using an inductive approach followed by deductive mapping to the RE-AIM framework. These themes included barriers to participation and engagement (Reach), acceptability of the intervention, perceived benefits for children and adolescents (Effectiveness), adoption within primary healthcare settings, facilitators and barriers to implementation, and maintenance.

### Acceptability

Providers expressed positive perceptions of the PROACT intervention and viewed it as a valuable and practical approach for supporting children and adolescents with mental health challenges. They reported that the training enhanced their skills and confidence in addressing mental health concerns and that the intervention was well received by both frontline providers and facility leadership.

“This is a program I’d recommend to any social worker … it’s very important to have such skills. Before, I didn’t know about thoughts, feelings and behavior triangle and how they affect each other, now I use it daily.” – Social Worker 4, Male

### Perceived benefits for children and adolescents

Providers reported observing meaningful improvements among children and adolescents who participated in the intervention. These changes included increased emotional awareness, improved management of anxiety symptoms, greater willingness to seek support, and enhanced functioning in everyday life. According to providers, participants learned practical coping skills that enabled them to manage emotional distress more effectively, particularly anxiety symptoms.

“Now they know, if I have anxiety, I can breathe, imagine I’m in Paris … in my happy place.” – Social Worker 2, Female

Providers also reported improvements in communication and help-seeking behaviors, noting that participants became more comfortable discussing emotional challenges with trusted adults and peers. Family members were also reported to notice positive changes among participating children.

“Changes you can see even from reports from their families, there’s one … you could just talk to her, she starts crying. Right now, she’s easy … keeps time.” – Social Worker 1, Female

### Adoption

The adoption of PROACT was facilitated by strong support from supervisors and facility leadership. Providers described receiving encouragement from their managers and viewed the intervention as addressing an important gap in child mental healthcare within primary healthcare settings.

“My in-charge was okay with it, this is a program that people, even my seniors, would embrace.” Social Worker 3, Male

In addition, providers reported increased confidence and competence in managing mental health concerns following the training. Some health workers observed noticeable changes in how trained social workers approached counseling and case management after learning the PROACT model.

“There is a change in her practice, she has a format now … she’s really empowered.” – Nurse In-charge, Female

Several providers reported extending the intervention principles beyond the target population, applying PROACT techniques in broader clinical encounters, including with adult patients experiencing anxiety and psychosocial distress.

“The therapy guide helps, if I follow this procedure, they can really help. I’ve used it with adults too … relaxation techniques help stressed adults.” – Social Worker 6, Female

### Implementation: facilitators and barriers to delivery

Providers generally described the implementation of PROACT as feasible within primary healthcare facilities. Several implementation facilitators were identified, including the structured intervention guide, regular supervision, and flexibility to tailor sessions to individual client needs. Providers particularly valued ongoing supervision, which helped maintain fidelity and supported clinical decision-making throughout intervention delivery.

“Before every session, you talk to your supervisor. Then you discuss how the session will be … the previous session, how it was and the next one … this one really keeps you in check. You’re not able to lose anything along the way.” – Social Worker 5, Male

Providers also emphasized the importance of adapting the pace of delivery according to each child’s needs and level of understanding.

“You just go according to your client’s pace, so that you make sure at the end of it all, he or she understands.” Social Worker 1, Female

Despite these facilitators, providers reported several implementation challenges. These included a lack of private counseling space, competing work responsibilities, interruptions from routine facility duties, scheduling difficulties, and challenges associated with session frequency and duration.

“We don’t have a private room here. This room, someone comes, knocks in … so I think that is one of the challenges.” – Social Worker 4, Male

“Sometimes I have to attend other clients in the hospital, because that is my primary responsibility here, and I get paid for that specifically,…. so if they need me to serve I cant say no, I have to go, and then If I get free time I see these patients in this programme” - Social Worker 3, Male

Providers also reported difficulties coordinating appointments because of competing demands on both provider and participant schedules.

“Sometimes it forced me to come over the weekends because the clients cannot come on weekdays.” – Social Worker 3, Male

Some providers felt that the one-week interval between sessions was too long, resulting in reduced retention of intervention content.

“A one week gap between sessions was a long period for the client to wait … sometimes the client will forget some of the things.” -Social Worker 2 Female

Others felt that younger participants occasionally struggled to remain engaged during longer sessions.

“The length of the sessions was the problem, because you find sometimes when you’re dealing with the children, the children get bored very fast for a one hour session.” – Social Worker 5 Male

### Barriers to participation and engagement

Service providers identified several barriers that influenced the ability of children and adolescents to participate in the PROACT intervention. One of the most prominent challenges was the competing survival needs faced by some participants. Social workers reported that some children prioritized immediate needs, such as obtaining food, over attending mental health sessions. In some cases, providers personally offered small amounts of money or food to facilitate participation.

“Sometimes someone comes in for a session for food … you have to give her maybe a cup of tea or give her money to go eat, I have to go to my pocket … give maybe a hundred bob … to motivate them to come.” – Social Worker 1, Female

School schedules also limited participation. Many children found it difficult to attend sessions during weekdays because of academic commitments, requiring providers to arrange alternative schedules, including weekend appointments.

“You find that they cannot come on weekdays … so you have to organize with them … sometimes come over the weekends.” – Social Worker 2, Female

Reaching students enrolled in boarding schools proved particularly difficult. Providers reported that these children often returned to school before completing the intervention and were therefore unable to benefit from the program.

“They were on holidays … then went back to school … boarding school students couldn’t attend, they miss the opportunity to get information and skills … a better way is to engage them in schools or during holidays.” – Social Worker 3, Male

### Maintenance

Providers expressed a strong intention to continue using PROACT beyond the study period, reflecting confidence in the intervention and a belief in its value for supporting children and adolescents experiencing emotional difficulties.

“Even after now I’ll continue doing it despite the end of the program, doing it with the few clients I have. So I’m grateful for this opportunity.” – Social Worker 1, Female

However, providers also identified factors that could affect long-term sustainability. A frequently mentioned concern was the limited number of trained personnel available to deliver the intervention, which could constrain future expansion and increase workload pressures on existing providers.

“If we can have some more people to deal with those cases then it can be great.”

Looking toward future scale-up, providers recommended extending PROACT beyond health facilities and into settings where young people spend most of their time, including schools, community spaces, faith-based settings, and potentially home environments. Social Worker 2, Female

“We can improve it by reaching out to more youth in the schools or in social places like churches or meetings so that we can engage them and help them.” – Social Worker 3, Male

## Discussion

This study examined changes in mental health symptoms among children and adolescents exposed to adverse childhood experiences following participation in the PROACT intervention delivered by trained social workers within routine primary healthcare settings in Nairobi, Kenya. Across the study period, participants demonstrated substantial reductions in symptoms of depression, anxiety, and post-traumatic stress. Improvements were observed consistently across both regression and non-parametric analyses, and most participants reported lower symptom scores at the end of the intervention compared to baseline. These findings suggest that PROACT can be delivered by non-specialist providers within routine service settings and may offer a promising approach for addressing the large unmet mental health needs of children and adolescents exposed to adversity in low-resource contexts (19, 20).

The findings are broadly consistent with a growing body of evidence supporting task-sharing approaches for child and adolescent mental healthcare in low- and middle-income countries (21, 22). Several studies have demonstrated that non-specialist providers, including lay counsellors, teachers, and social workers, can successfully deliver structured psychosocial interventions and support improvements in common mental health problems among young people (20, 21). Similar improvements in depression, anxiety, and trauma-related symptoms have been observed in studies evaluating evidence-based psychosocial interventions delivered by non-specialist providers, including the Common Elements Treatment Approach (CETA) (22) and Problem Management Plus (PM+) (23, 24). These findings reflect on the current growing recognition that the shortage of mental health specialists should not prevent access to evidence-based mental healthcare when appropriate training, supervision, and implementation support systems are available (13, 22).

An important feature of PROACT is its transdiagnostic and modular design. Unlike interventions developed to target a single mental disorder, PROACT addresses common emotional and behavioural difficulties through a flexible package of psychoeducation, relaxation, problem-solving, behavioural activation, and cognitive coping strategies. This approach shares similarities with interventions such as CETA (23), which uses common therapeutic elements across multiple mental health conditions, and offers potential advantages in routine primary care settings where children often present with overlapping symptoms rather than a single diagnosis. In many resource-constrained settings, service providers have limited time, training opportunities, and specialist support. Interventions that can address multiple psychological difficulties within a single framework may therefore be particularly valuable (21, 22). The positive symptom changes observed across depression, anxiety, and PTSD domains in this study provide preliminary support for the potential utility of such an approach.

The implementation findings further strengthen the relevance of PROACT within routine healthcare systems. Providers reported that the intervention was acceptable, practical, and useful in their day-to-day work. Social workers described increased confidence in identifying and responding to mental health concerns and reported applying intervention skills beyond the study population. Similar findings have been reported in other task-sharing initiatives where provider confidence, supervision, and structured intervention guides have been identified as important facilitators of implementation. The positive perceptions expressed by providers and facility leadership suggest that PROACT may fit well within existing service delivery structures and could potentially be integrated into broader child and adolescent mental health services.

At the same time, the qualitative findings highlight important contextual realities that may influence implementation success. Poverty-related barriers, competing educational demands, lack of private counselling space, staff shortages, and competing clinical responsibilities were commonly reported. These challenges have also been documented in mental health implementation studies across sub-Saharan Africa and reflect broader health system constraints rather than intervention-specific weaknesses (23). The findings therefore suggest that successful scale-up of PROACT will likely require attention not only to provider training but also to organizational readiness, protected counselling spaces, supervision systems, and mechanisms to support participation among vulnerable children who face competing social and economic priorities (24–26).

The findings of this study should, however, be interpreted with caution. Although significant symptom reductions were observed following participation in the intervention, the study employed a single-group pre-post design without a comparison group. Consequently, it is not possible to determine with certainty whether the observed improvements were attributable solely to PROACT. Alternative explanations, including natural recovery, regression to the mean, repeated assessment effects, increased attention from providers, or other unmeasured influences, cannot be excluded. The mixed-effects regression analyses accounted for repeated observations within participants but did not provide a counterfactual against which intervention outcomes could be compared. Accordingly, the findings are best interpreted as preliminary evidence of symptom improvement following participation in PROACT rather than definitive evidence of intervention effectiveness.

Selection processes may also have influenced the findings. Children were recruited using convenience sampling, while intervention providers and participating facilities were purposively selected. Consequently, the study sample may not fully represent the broader population of children and adolescents exposed to ACEs in Nairobi. Although six participants withdrew before completing the intervention, most withdrawals occurred when participants returned to boarding school following the holiday period. No meaningful differences were observed in baseline characteristics or symptom severity between participants who completed the intervention and those who withdrew. Nevertheless, the small sample size limits our ability to fully exclude the possibility of attrition-related bias.

### Strengths and limitations

This study has several strengths. First, it evaluated the delivery of a structured psychosocial intervention within routine primary healthcare settings rather than under highly controlled research conditions, increasing its practical relevance for real-world service delivery. Second, the intervention was delivered by social workers who are already embedded within existing health systems, enhancing the applicability of findings to settings facing severe shortages of specialist mental health providers. Third, the mixed-methods design enabled examination of both participant outcomes and implementation experiences, providing a more comprehensive understanding of intervention feasibility, acceptability, and delivery challenges.

Several limitations should also be considered. The absence of a comparison group limits causal inference and prevents conclusions regarding intervention effectiveness. The relatively small sample size reduces statistical power and limits generalizability to other populations and settings. Participants were recruited from selected facilities within Nairobi County and may not represent children and adolescents living in other regions or contexts. Importantly, the study relied on baseline and endline assessments only, limiting our ability to determine whether the observed improvements were maintained over time. In addition, qualitative findings were based on a small number of key informant interviews and primarily reflect provider perspectives rather than the experiences of children and caregivers themselves. Another limitation relates to the delivery of the intervention by social workers rather than specialist mental health professionals. Althouh, the task-sharing stratergy offers a promising solution for expanding access to mental health services in low-resource settings, differences in training, clinical experience, and competency may influence intervention fidelity and the ability to identify and manage complex or high-risk mental health presentations. Although social workers received structured training and ongoing supervision throughout the study, variations in intervention delivery may have occurred and could have influenced participant outcomes. Future studies should consider more intensive supervision, ongoing fidelity monitoring, and periodic booster training sessions to enhance consistency and uniformity in intervention delivery across providers. The findings nevertheless provide important preliminary evidence supporting further evaluation of PROACT. Future studies should employ randomized controlled designs or other rigorous comparative approaches to establish effectiveness and strengthen causal inference. Longer follow-up periods are needed to assess the durability of symptom improvements, while larger implementation studies should examine intervention performance across diverse geographic and service settings. Future research should also explore strategies for improving access among children attending boarding schools, reducing implementation barriers within health facilities, and integrating PROACT into schools and community-based platforms where children and adolescents routinely interact.

## Conclusion and summary of findings

Our study aimed to pilot the PROACT intervention, which was adapted for Social Worker with no specialized knowledge in mental health. Specifically, the goal was to determine whether Social Workers could manage mild to moderate symptoms of anxiety, depression, and post-traumatic stress disorder (PTSD) among children and adolescents with a history of adverse childhood experiences living in informal settlements in Nairobi County. The PROACT intervention is one of the novel, culturally adapted psychological interventions initially designed for children and adolescents living with HIV in Kenya. To the best of our knowledge, this is the first study to adapt and implement this intervention among children with a history of adverse childhood experiences in Nairobi.

Our study found a significant reduction in depressive, anxiety, and PTSD symptoms following the PROACT intervention, with a moderate magnitude of change from baseline scores. These findings suggest that the PROACT intervention, when adapted for community health providers without specialized mental health training, can be considered a promising psychological approach for use in low-income settings with limited availability of mental health professionals.

## Data Availability

The original contributions presented in the study are included in the article/**Supplementary Material**. Further inquiries can be directed to the corresponding author.
